# Taurine as an Essential Neuromodulator during Perinatal Cortical Development

**DOI:** 10.3389/fncel.2017.00328

**Published:** 2017-10-24

**Authors:** Werner Kilb, Atsuo Fukuda

**Affiliations:** ^1^Institute of Physiology, University Medical Center, Johannes Gutenberg University of Mainz, Mainz, Germany; ^2^Department of Neurophysiology, Hamamatsu University School of Medicine, Hamamatsu, Japan

**Keywords:** cerebral cortex, subplate, Cajal–Retzius cells, GABA receptors, glycine receptors, migration, rodent, review

## Abstract

A variety of experimental studies demonstrated that neurotransmitters are an important factor for the development of the central nervous system, affecting neurodevelopmental events like neurogenesis, neuronal migration, programmed cell death, and differentiation. While the role of the classical neurotransmitters glutamate and gamma-aminobutyric acid (GABA) on neuronal development is well established, the aminosulfonic acid taurine has also been considered as possible neuromodulator during early neuronal development. The purpose of the present review article is to summarize the properties of taurine as neuromodulator in detail, focusing on the direct involvement of taurine on various neurodevelopmental events and the regulation of neuronal activity during early developmental epochs. The current knowledge is that taurine lacks a synaptic release mechanism but is released by volume-sensitive organic anion channels and/or a reversal of the taurine transporter. Extracellular taurine affects neurons and neuronal progenitor cells mainly via glycine, GABA(A), and GABA(B) receptors with considerable receptor and subtype-specific affinities. Taurine has been shown to directly influence neurogenesis *in vitro* as well as neuronal migration *in vitro and in vivo*. It provides a depolarizing signal for a variety of neuronal population in the immature central nervous system, thereby directly influencing neuronal activity. While in the neocortex, taurine probably enhance neuronal activity, in the immature hippocampus, a tonic taurinergic tone might be necessary to attenuate activity. In summary, taurine must be considered as an essential modulator of neurodevelopmental events, and possible adverse consequences on fetal and/or early postnatal development should be evaluated for pharmacological therapies affecting taurinergic functions.

## Introduction

The aminosulfonic acid taurine (2-aminoethanesulfonic acid) is among the most abundant organic molecules in the human body, including the CNS, and has been attributed to a variety of physiological functions (for review Huxtable, 1989; Lambert et al., 2015; Oja and Saransaari, 2015). Taurine is involved in cell volume regulation (Solis et al., 1988; Lambert, 2004), mitochondrial translation (Suzuki et al., 2002), and Ca^2+^ homeostasis (Chen et al., 2001; El Idrissi, 2008b). Taurine has been suggested to enhance the stability of membranes and directly stabilizes membrane proteins (You and Chang, 1998; Roychoudhury et al., 2013). It modulates inflammation (Marcinkiewicz and Kontny, 2014) and has been shown to reduce apoptosis in a variety of tissues, including the CNS (Taranukhin et al., 2008; Ramos-Mandujano et al., 2014). In addition, taurine possesses an antioxidant effect that, as taurine itself only poorly scavenges radicals (Martincigh et al., 1998), is mediated via indirect effects (Schaffer et al., 2009). In line with this, a variety of studies demonstrated that taurine protects the brain from ischemic or traumatic insults (Sun et al., 2012, 2015; Menzie et al., 2013), including models of perinatal asphyxia (Zhu et al., 2016). Also, taurine is an endogenous agonist of glycine and γ-aminobutyric acid (GABA) receptors (Albrecht and Schousboe, 2005). Therefore, taurine is considered as an endogenous neuromodulator providing an inhibitory effect on the mature CNS. In accordance with this actions, animal studies reported anticonvulsive actions of taurine (El Idrissi et al., 2003; El Idrissi and L’Amoreaux, 2008), which, however, were not completely replicated in humans (reviewed in Oja and Saransaari, 2013). In line with an inhibitory action in the spinal cord, taurine also has as a considerably antinociceptive effect (Pellicer et al., 2007; Terada et al., 2011; Hara et al., 2012). In addition, taurine improves different *in vitro* correlates of memory formation (Chepkova et al., 2002; del Olmo et al., 2003; Sergeeva et al., 2003) and accordingly augments learning and memory (El Idrissi, 2008a; Neuwirth et al., 2013).

In the immature brain, the taurine concentration is at least 3 times higher than in the adult nervous system, with a considerable downregulation after the first postnatal week in rodents (Huxtable, 1989; Benitez-Diaz et al., 2003). The stimulated taurine release is also significantly larger in immature brains than in adult brains (Oja and Saransaari, 1995). Both observations suggest that taurine may play a particular important role during neuronal development. This suggestion was substantiated by the observations that the development of the visual cortex and the cerebellum was impaired in taurine deficient kitten (Sturman et al., 1985; Palackal et al., 1986). Since these seminal findings of John Sturman, additional studies have been published supporting the hypothesis that taurine is critically involved in a series of neurodevelopmental events. In the following, we like to (i) describe the properties of taurine as neuromodulator in detail and (ii) present recent findings that demonstrate the involvement of taurine on differential neurodevelopmental events.

## Taurine Release Mechanisms and Taurine Receptors

**FIGURE 1:**
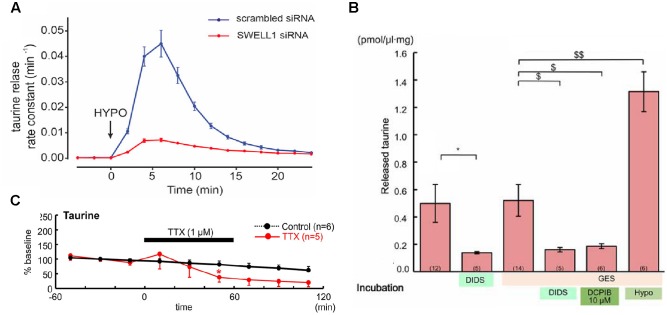
Taurine release pathways. **(A)** The taurine release from HeLa cells after hypoosmotic stimulation was massively attenuated if expression of the volume-regulated anion channel SWELL1 was suppressed (with permission from Qiu et al., 2014). **(B)** The taurine release from embryonic neocortical slices loaded with 10 mM taurine was not affected by the TauT inhibitor GES, could be blocked the unspecific anion channel blocker DIDS or by DCPIB, a selective blocker of volume-regulated anion channels, and was stimulated by hypoosmotic stimulation (hypo), suggesting that taurine efflux was mainly mediated by volume-regulated anion channels (^∗^ and ^$^ represent *P* < 0.05, ^$$^ indicate *P* < 0.01, with permission from Furukawa et al., 2014). **(C)** Suppression of electrical activity attenuated the spontaneous taurine release from tangential slices of early postnatal rat neocortex, suggesting the existence of a constitutive, activity-dependent taurine release (modified with permission from Qian et al., 2014).

For classical neurotransmitter systems, the existence of vesicular transporters, synaptic release mechanisms, and specific receptors has been described. Taurine differs from these substances in some points. First, to our knowledge, no vesicular transports systems for taurine have been identified and taurine release seems to be mainly independent of Ca^2+^ influx (Kamisaki et al., 1996). The main release pathways for taurine are therefore volume-sensitive organic anion channels (**Figure 1A**; Qiu et al., 2014; Voss et al., 2014) and/or a reversal of the TauT (SLC6A6; Saransaari and Oja, 2000c). However, in the immature cortex, taurine release seems to occur mainly via volume-sensitive organic anion channels (**Figure 1B**; Furukawa et al., 2014). Although in the immature nervous system Ca^2+^-dependent taurine release has been reported (Saransaari and Oja, 1999), later studies demonstrated that this effect is probably secondary to the vesicular release of other neurotransmitters that modulate taurine release (Saransaari and Oja, 2000c). The basal, unstimulated taurine release in the early postnatal CNS has been found to be lower than in the adult CNS (Saransaari and Oja, 2006). However, a variety of stimuli can trigger taurine release in the immature nervous system including volume changes (Oja and Saransaari, 1995), hypoosmotic stimulation (Furukawa et al., 2014), ischemia (Saransaari and Oja, 1999), glutamate, via NMDA, AMPA, and metabotropic receptors (Saransaari and Oja, 1991, 2000a, 2003), and adenosine (Saransaari and Oja, 2000b). In addition, a constitutive taurine release by electrical activity has been observed in the immature neocortex (**Figure 1C**; Qian et al., 2014).

**FIGURE 2:**
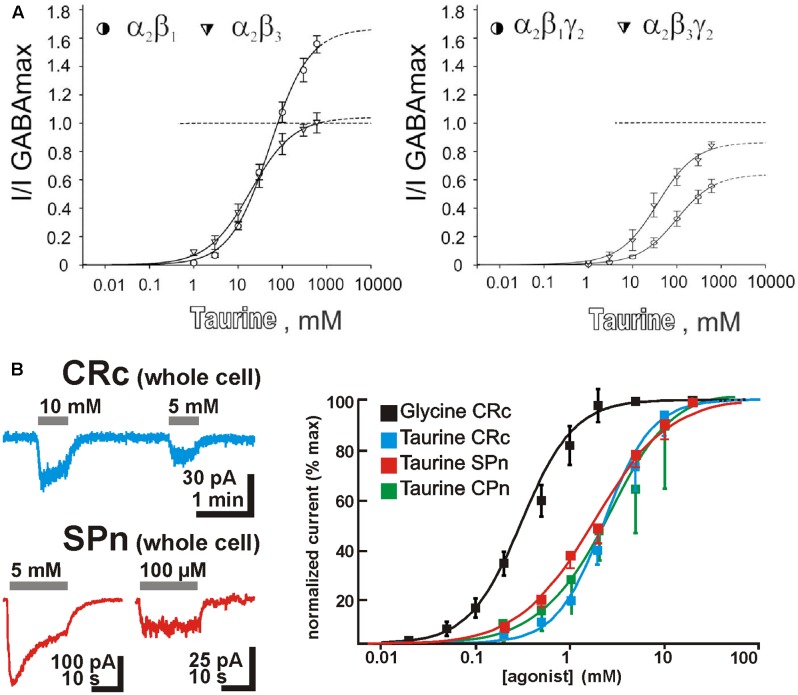
Properties of taurine receptors. **(A)** Taurine affinities and maximal taurine currents critically depend on the subunit composition of GABA_A_ receptors (modified with permission from Kletke et al., 2013). **(B)** Typical membrane currents and dose–response curves of taurine on glycine receptors for different neuron populations in the immature neocortex revealed that taurine is a low-affinity agonist with comparable affinities in CRc (blue), SP neurons (SPn, red), and neurons from the CP and developing layers (CPn, green) (modified with permission from Kilb et al., 2002, 2008).

It is regularly stated, that taurine is a partial, low-affinity agonist on GABA_A_ receptors (Albrecht and Schousboe, 2005). However, recent studies demonstrated that the action of taurine depends critically on the subunit composition of these receptors (**Figure 2A**). GABA_A_ receptors are heteropentameric complexes composed from total 19 subunits (Farrant and Kaila, 2007). For α1 and α2 containing receptors lacking γ2 subunits, taurine seems to be a full agonist, while addition of γ2 subunits to the pentameric complex reduced the amplitude of maximal taurine currents (Kletke et al., 2013). Interestingly, on α2/β1 receptors, GABA acts as superagonist, albeit with a low affinity (Kletke et al., 2013). For all receptors containing α1 or α2 subunits, a rather low taurine affinity above an EC_50_ value of 10 mM was observed (Dominguez-Perrot et al., 1996; Kletke et al., 2013). On the other hand, GABA_A_ receptors assembled from α4, β2, and δ subunits, which mediate extrasynaptic currents, have a rather high taurine affinity with an EC_50_ of 57 μM (Ahring et al., 2016) and taurine mediates a larger current than GABA by these receptor subtypes. Accordingly, relatively low taurine concentrations of 10–100 μM mediate a substantial tonic current in thalamic neurons expressing these receptor subtypes (Jia et al., 2008). GABA_A_ receptors containing α6, β2, and δ subunits, which mediate extrasynaptic currents in the cerebellum, express a partially high-binding state with a rather high taurine affinity (EC_50_ ca. 6 μM, Hadley and Amin, 2007). Taurine is also a partial agonist of the ionotropic GABA_C_ (ρ subunit-containing GABA_A_) receptor, albeit again with a rather low affinity (EC_50_ ca. 5 mM, Ochoa-de la Paz et al., 2008). However, ρ subunit-containing hybrid ionotropic GABA receptors (which combine properties of GABA_A_ and GABA_C_ receptors) seem to be more sensitive to taurine and mediate considerable tonic currents at submillimolar taurine concentrations (Chesnoy-Marchais, 2016). An interesting observation is that micromolar taurine concentrations can massively enhance tonic GABAergic currents, which suggest that extrasynaptic GABA and taurine may act synergistically (Ochoa-de la Paz et al., 2008). In summary, these observations indicate that specific subunit compositions of GABA_A_ receptors can assemble high-affinity taurine receptors and indicate that even μM concentrations of taurine can be sufficient to mediate a physiologically relevant activation of ionotropic GABA_A_ receptor subpopulations. Thus, taurine can be considered as a full agonist with a reasonable affinity for distinct, physiologically relevant GABA_A_ receptor subtypes.

In addition to ionotropoic GABA_A_ and GABA_C_ receptors, taurine can also interact with metabotropic GABA_B_ receptors. Baclofen and GABA-replacement experiments suggested that taurine can activate GABA_B_ receptors as a rather high-affinity ligand in the μM range (Kontro and Oja, 1990). In line with this, taurine acts as chemoatractant for migrating neurons via a saclofen-sensitive interaction with GABA_B_ receptors at a concentration of 1 μM (Behar et al., 2001), illustrating the physiological role of GABA_B_ receptors as high-affinity receptors for taurine.

Finally, taurine is also a partial agonist of glycine receptors (Albrecht and Schousboe, 2005). Glycine receptors are composed from four α and one β subunits and form α homomeric or α/β heteromeric receptors (Betz et al., 1999). α1 homomeric receptors have a low taurine affinity with an EC_50_ of 3.7 mM, while α2 homomeric receptors demonstrate a slightly higher taurine affinity with an EC_50_ of 2.2 mM (Schmieden et al., 1992). In immature neocortical neurons of the rat, where most probably α2/β heteromeric glycine receptors were expressed, the taurine affinity of these receptors was in the range between 1.1 and 2.4 mM (**Figure 2B**; Flint et al., 1998; Okabe et al., 2004; Kilb et al., 2008). In summary, these results demonstrate that taurine can be considered as low-affinity agonist for glycine receptors.

In addition to the well-described actions of taurine on GABA and glycine receptors, a few studies also identified NMDA receptors, a ionotropic glutamate receptor subtype, as putative targets of taurine (Suarez and Solis, 2006; Chan et al., 2013). As low as 100 μM taurine is sufficient to augment NMDA-dependent actions on hippocampal fiber volleys, but not postsynaptic NMDA receptors (Suarez and Solis, 2006), suggesting that presynaptic NMDA receptors are potentiated by taurine. In contrast, postsynaptic NMDA receptors seem to be rather inhibited by taurine, although at substantial higher concentration in the mM range (Chan et al., 2014). It was speculated that this postsynaptic inhibition is mediated via an interaction with the allosteric glycine binding site of NMDA receptors (Chan et al., 2014) and depends on NR2B subunits (Chan et al., 2015).

Regarding its nonsynaptic release pathways, taurine can be considered rather as endogenous neuromodulator than as classical neurotransmitter. Thus, it is comparable to other endogenous neuromodulators of GABA receptors, like neurosteroids (Belelli and Lambert, 2005) or the putative endozepine diazepam-binding inhibitor (Christian et al., 2013), of glycine receptors, like Zn^2+^ (Hirzel et al., 2006), and of NMDA receptors, like D-Serine (Henneberger et al., 2012).

With respect to the observations that different receptors/receptor subtypes have a wide range of affinities for taurine, it is of course essential to know the interstitial taurine concentration in the immature brain. Unfortunately, direct measurements of extracellular taurine concentrations in the immature brain *in vivo* have not been published. From (i) the taurine concentration of 25 μM measured in the mature CNS under zero-flow conditions by means of microdialysis probes (Molchanova et al., 2004) and (ii) the observation that the total taurine concentration in the immature CNS during the first postnatal week is at least 3 times larger than in the adult CNS (Benitez-Diaz et al., 2003), it can be assumed that the interstitial taurine concentration can reach values above 75 μM. On the other hand, loading experiments suggested that the taurine concentration could be as high as 1 mM in the embryonic neocortex (Furukawa et al., 2014).

## Neurotransmitters and Neuronal Activity Influence Corticogenesis

### A Short Summary of Neurodevelopmental Events

During development, neurons undergo specific steps of maturation, including neurogenesis, neuronal migration, differentiation, and pruning. The cerebral cortex of vertebrates originates from the two telencephalic vesicles. The earliest neurons generated in the neuroepithelium of this vesicles form the PPL (Angevine and Sidman, 1961). The proliferative zones close to the ventricular surface, termed VZ and SVZ, subsequently continue to generate neurons, which migrate along radial glial processes toward the pial surface and split the PPL into the superficial MZ and the underlying SP. In addition, GABAergic neurons generated in the ganglionic eminences reach the immature neocortex by tangential migration (for review Marín and Rubenstein, 2001). Both MZ and SP are populated by specific neuronal populations, called CRc and SP neurons, respectively (Luhmann et al., 2009; Kirischuk et al., 2014). In between these two transient layers, the CP establishes, which will later develop into layers II to VI of the neocortex (see Bystron et al., 2008 for review). CRc in the MZ play an essential role for cortical lamination and disappear later during development, generating the cell sparse layer I (for review Kirischuk et al., 2014). SP neurons guide the projection of thalamocortical afferents and are transient synaptic targets of these axons before their final targets in layer IV appear (for review Kanold and Luhmann, 2010). The different neuronal populations that build up the mature neocortex later differentiate to form the right connectivity. Subsequently not only a considerable amount of neurons, but also ineffective connections are removed (for review Nikolic et al., 2013). A variety of reports demonstrated that neuronal activity and various neurotransmitters influence cortical development at different levels, ranging from early events as neurogenesis or migration to the establishment of fine-scale neuronal connectivity (for reviews Owens and Kriegstein, 2002; Spitzer, 2006; Wang and Kriegstein, 2009; Luhmann et al., 2016; Khazipov and Milh, 2017).

Immature neuronal activity starts with spontaneous calcium waves that have been observed in mouse neocortical slices already at early embryonic stages in both the VZ (Owens et al., 2000) and the early CP (Corlew et al., 2004). This early *in vitro* activity was replaced by a distinct set of large-scale network events, beginning with spontaneous and glutamate receptor dependent early network oscillations (Garaschuk et al., 2000), which were followed by giant depolarizing potentials a few days later, characterized by their dependence on depolarizing GABA_A_ receptor-mediated transmission (Allène et al., 2008). *In vivo* recordings confirmed that spontaneous discontinuous activity occurs already in the early postnatal neocortex (e.g., Khazipov et al., 2004; Hanganu et al., 2006; Yang et al., 2009; Colonnese and Khazipov, 2010). Already at birth local and short network oscillations, termed spindle bursts, in a frequency range of 10–20 Hz occurred, which were a few days later complemented by faster gamma oscillations with a frequency of 30–40 Hz (Khazipov et al., 2013; Yang et al., 2016). This discontinuous activity is generated in the sensory periphery, often independent of physical stimuli, subcortical regions, and in the immature neocortex itself (for review Luhmann et al., 2016; Khazipov and Milh, 2017).

These different patterns of neuronal activity play important roles for various physiological processes during neuronal development. Proliferation in the VZ is directly influenced by spontaneous calcium waves and spontaneous activity projected from the sensory periphery (Weissman et al., 2004; Bonetti and Surace, 2010). Spontaneous rhythmic intracellular calcium waves control neuronal migration (Komuro and Kumada, 2005). In addition, the rate of apoptosis as programmed cell death, which is an essential factor structuring the neocortical circuits, also directly depends on electrical activity (for review Blanquie et al., 2017a). And finally, spontaneous neuronal activity influences growth and differentiation of neuronal dendrites and axonal projections (for reviews Yamamoto and López-Bendito, 2012; Luhmann et al., 2016) and contributes to the formation of topographic maps (for reviews Hanganu-Opatz, 2010; Assali et al., 2014; Luhmann and Khazipov, 2017). While for many of these activity patterns, a vesicular release of GABA and glutamate is essential, recent studies identified taurine as a factor that contribute to GABAergic effects on neuronal development and also directly controls activity levels in the immature CNS as follows.

### Taurine Affects Corticogenesis

The neurogenesis in the VZ and SVZ is influenced by GABA_A_ receptors (LoTurco et al., 1995). Activation of GABA or glycine receptors also directly controls neuronal migration (for review Luhmann et al., 2015). Activation of GABA_A_ receptors induces apoptosis in CRc (Blanquie et al., 2017b), but promotes neuronal differentiation and synaptogenesis in principal cells and interneurons (Maric et al., 2001; Meier et al., 2003; see Wang and Kriegstein, 2009 for review). However, it should be noted in this respect that none of the mentioned studies unequivocally identified GABA as the endogenous neurotransmitter mediating these actions, but only demonstrated that GABA_A_ receptors are required. In fact, it has been shown that (i) neuronal migration is influenced by GABA_A_ receptors even in the absence of synaptic neurotransmitter release (Manent et al., 2005) and (ii) tonic GABA_A_ receptor mediated currents in embryonic neurons are unaffected by a massive GABA depletion, but sensitive to conditions enhancing extracellular taurine levels (Furukawa et al., 2014), allowing the speculation that taurine may contribute as endogenous agonist to the reported effects. With respect to the GABAergic and glycinergic actions, it must be considered that both neurotransmitters mediate a depolarizing action in the immature CNS, due to a high expression rate of the chloride loader NKCC1 and a low expression of the chloride extruder KCC2 (Yamada et al., 2004; Watanabe and Fukuda, 2015). Taurine is directly involved in this process, as it mediates an inhibition of KCC2 function via phosphorylation in immature neurons, thus maintaining a depolarizing action of GABA_A_ or glycine receptors (Inoue et al., 2012). In the following, we will summarize the action of taurine on distinct neuronal subpopulations and discuss how this interaction can interfere with neuronal development.

## Action of Taurine on Identified Neuronal Populations in the Developing Brain

### Neural Stem Cells

In the SVZ of the cortex, the activation of GABA_A_ receptors significantly reduced neurogenesis (LoTurco et al., 1995; Haydar et al., 2000). The observation that pharmacological inhibition of GABA_A_ receptors enhances the proliferation (LoTurco et al., 1995) indicates that an intrinsic agonist of GABA_A_ receptors controls neurogenesis. In the VZ, however, activation of GABA_A_ receptors increases proliferation (Haydar et al., 2000), probably by preventing neuroblast from exiting the proliferative cycle toward G0 phase. Since the proliferation of neuroblasts in the postnatal SVZ is limited by a nonsynaptic release of GABAergic agonists (Liu et al., 2005), it is intriguing to speculate that taurine contributes to these effects. Neuroepithelial stem cells directly contact cerebrospinal fluid (Lehtinen and Walsh, 2011; Lehtinen et al., 2011; Chau et al., 2015), which at least during early developmental stages subsequently to neural tube closing is consisting mainly of amniotic fluid, in which taurine is accumulated from maternal blood (Sturman et al., 1977; Sturman, 1981).

**FIGURE 3:**
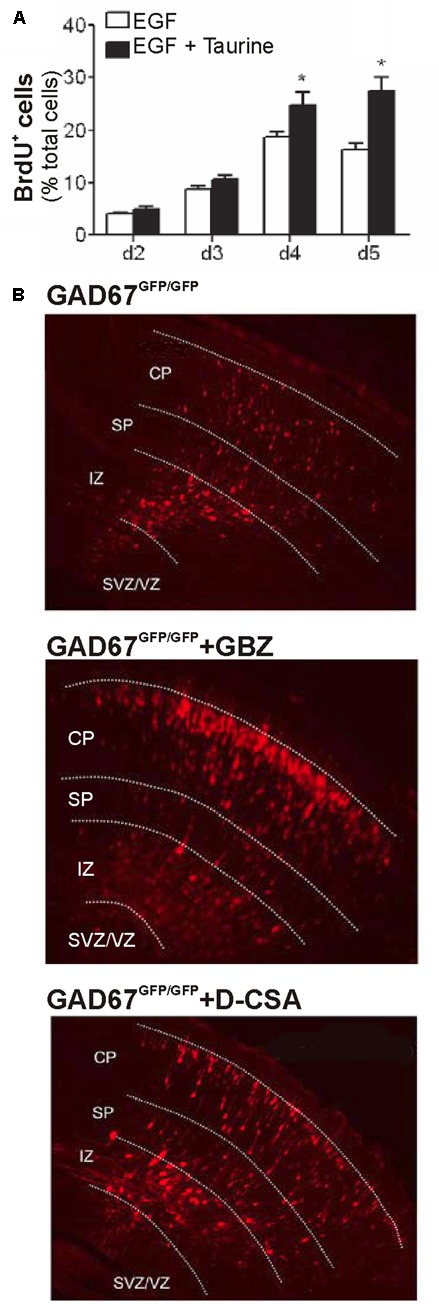
Effect of taurine on proliferation and migration. **(A)** Taurine (10 mM) enhances the fraction of BrdU positive neurons in mouse neurospheres after 4–5 days in culture, indicating that taurine promotes proliferation (^∗^ indicate *P* < 0.05, with permission from Hernandez-Benitez et al., 2010). **(B)** Radial migration of RFP-labeled neurons in the substantially GABA-depleted GAD67-GFP mice can be enhanced by inhibition of GABA_A_ receptors with gabazine (middle image) and by inhibition of maternal taurine synthesis with D-cysteinate (D-CSA, lower image), indicating that taurine acting of GABA_A_ receptors modulate radial migration *in vivo* (modified with permission from Furukawa et al., 2014).

Recently it has been demonstrated that 10 mM taurine indeed increases the proliferation of mice embryonic progenitor cells *in vitro* (Hernandez-Benitez et al., 2010; **Figure 3A**). Similar effects were also found for human neuronal precursor cells, where proliferation is also enhanced by millimolar amounts of taurine, albeit it is not clear whether this process requires GABA_A_ or glycine receptors (Hernandez-Benitez et al., 2013). For hippocampal progenitors, it has also been demonstrated that 100 μM taurine augment proliferation (Shivaraj et al., 2012). In the mouse cochlea, millimolar taurine concentrations enhance proliferation of stem cells, but augment only the number of glutamatergic neurons, while the number of GABAergic neurons descending from cochlear stem cells is decreased (Wang et al., 2015). These results suggest that taurine can also provide a signal that determines the composition of neuronal elements within a circuitry. A similar observation was also made in the retina, where 100 μM taurine acting via glycine receptors stimulate the differentiation of retinal progenitor cells toward rod photoreceptors (Altshuler et al., 1993; Young and Cepko, 2004).

Intriguingly, electrophysiological experiments failed to reveal taurine-induced membrane currents in cells of the VZ (Flint et al., 1998), suggesting that these cells probably did not express taurine-sensitive glycine receptors. However, as from this publication, it is not clear whether the taurine experiments were performed in the presence of GABAergic antagonists, taurine probably affect neuroblasts exclusively via GABA_A_ receptors functionally expressed in VZ neuroblasts (LoTurco et al., 1995; Ma and Barker, 1995; Tochitani et al., 2010). On the other hand, it should also be considered that activation of GABA_A_ and/or glycine receptors can also influence proliferation indirectly, e.g., via interactions with bFGF or brain-derived neurotrophic factor (BDNF) release (Berninger et al., 1995; Antonopoulos et al., 1997).

Interestingly, taurine can also enhance adult neurogenesis under both *in vitro* and *in vivo* conditions (Hernandez-Benitez et al., 2012; Ramos-Mandujano et al., 2014; Gebara et al., 2015). This enhanced adult neurogenesis may underlie the beneficial effect of chronic taurine administration on various learning paradigms in adult mice (El Idrissi, 2008a; Neuwirth et al., 2013; Kim et al., 2014), although it should be noted that taurine can also directly affect *in vitro* correlates of memory formation (Chepkova et al., 2002; del Olmo et al., 2003; Sergeeva et al., 2003).

### Migrating Neurons

Already Sturman et al. (1985) suggested that in taurine deficient kitten neuronal migration in the cerebellum was hampered. In the visual cortex of these animals, clear indications for massively impaired migration were observed (Palackal et al., 1986). Taurine induced small inward currents in putatively migrating neurons of the rat cortical IZ at E19 via an activation of glycine receptors (Flint et al., 1998), whereas the taurinergic currents in clearly identified radially migrating neurons of the mouse neocortex were exclusively mediated via GABA_A_ receptors (Furukawa et al., 2014). In addition, it has been shown that GABA_A_ receptors (Barker et al., 1998; Heck et al., 2007), GABA_B_ receptors (Behar et al., 2001), GABA_C_ receptors (Denter et al., 2010), and glycine receptors (Nimmervoll et al., 2011), all possible targets of taurine, affect neuronal migration. Regarding the GABA_B_ receptors, Behar et al. (2001) directly demonstrated that taurine acts as potent chemoatractant for migrating neurons via an interaction with GABA_B_ receptors.

In dissociated cultures of cerebellar granule cells, taurine depletion indeed attenuated neuronal migration (Maar et al., 1995), which replicates the *in vivo* observation of Sturman et al. (1985) in the cerebellum of taurine deficient kitten. Recent *in vivo* studies by Furukawa et al. (2014) utilized GABA-deficient mice to specify the role of taurine for radial migration. Interestingly, they observed that radial migration was not significantly affected in a homozygous GAD-67 mouse, in which the GABA content was reduced to 12.7% of the wild-type level. However, inhibition of GABA_A_ receptors accelerated radial migration in this GAD-67 deficient mouse to a similar extent as in wild-type animals (Furukawa et al., 2014; **Figure 3B**), suggesting that GABA is not required as endogenous ligand for the GABA_A_ receptors regulating migration. This suggestion was substantiated by the findings that (i) the tonic currents in migrating neurons were unaffected by GABA depletion but (ii) enhanced in the presence of the TauT blocker GES, and (iii) attenuated after blockade of taurine synthesis with D-cystein (Furukawa et al., 2014; **Figure 3B**). And in line with these *in vitro* experiments, indicating that taurine is probably the most relevant endogenous agonist of extrasynaptic GABA_A_ receptors, depletion of taurine by maternal D-cystein administration accelerated migration in both wild-type and homozygous GAD-67 mice *in vivo* (Furukawa et al., 2014). In summary, these experiments provide mechanistic evidences that taurine is indeed a major endogenous modulator of radial migration.

### Cajal–Retzius Cells

In CRc, taurine-induced inward currents were mediated by glycine receptors with a low affinity (EC_50_ = 2.4 mM, Kilb et al., 2002; **Figure 2B**). In line with the high intracellular Cl^-^ concentration in these cells (Achilles et al., 2007), activation of glycine receptors mediate a membrane depolarization in CRc (Kilb et al., 2002). Qian et al. (2014) were able to demonstrate that a strong electrical stimulation indeed stimulates the release of taurine and GABA, while glycine was not released. Voltage sensitive dye imaging revealed that such strong electrical stimulation induced a rapidly propagating wave of depolarization in the MZ of tangential slices. This propagating activity was insensitive to glutamate receptor blockade, but was partially attenuated by the application of either GABAergic or glycinergic antagonist, while application of both GABAergic and glycinergic antagonists completely abolished this activity (Qian et al., 2014). Because microdialysis experiments performed in this study demonstrate that glycine was not released (Qian et al., 2014), these results suggest a contribution of taurine. Since this propagating activity was observed at the pial surface of tangential slices in the MZ, most probably CRc contribute to this activity. Accordingly, whole-cell recordings from CRc revealed that these electrical stimuli induced a strong inward current that was also insensitive to glutamatergic antagonists, only partially reduced by GABAergic and glycinergic antagonists and completely blocked if GABAergic and glycinergic antagonists were combined (Qian et al., 2014). Since taurine was not localized in the presynaptic structures and glycine was not released after electrical stimulation (Qian et al., 2014), these results suggest that, although a substantial portion of this tangentially propagating activity transients is mediated by synaptic release of GABA, taurine acting on GABA_A_ and glycine receptors in CRc also considerably contributed to the propagation of activity in the MZ in a neuromodulatory fashion.

### Neurons in the Cortical Plate or Developing Cortical Layers

**FIGURE 4:**
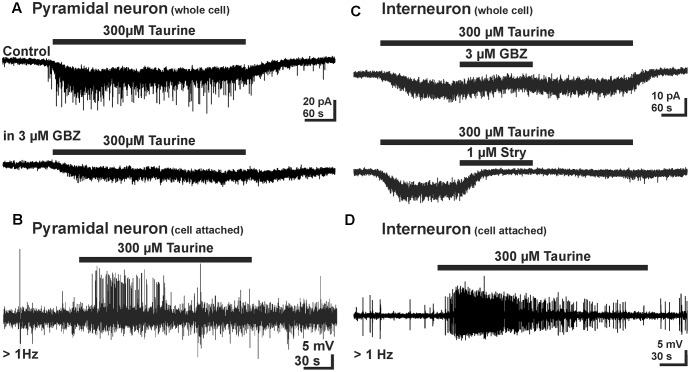
Effect of taurine on GABAergic networks in early postnatal mouse neocortex. **(A)** In pyramidal neurons taurine induced a tonic inward current and increased the frequency of GBZ-sensitive GABAergic PSCs. **(B)** Cell-attached recordings demonstrating that GABAergic PSCs enhance action potential frequency, suggesting that the taurine-induced GABAergic PSCs are excitatory. **(C)** In GABAergic interneurons taurine induced an inward-current that was relatively insensitive to GBZ, but suppressed by strychnine, indicating that taurine acts mainly via glycine receptors in this cell type. **(D)** Cell-attached recordings from GABAergic interneurons demonstrate that the taurine-induced inward current enhances action potential frequency, suggesting that the taurine is an excitatory neuromodulator in immature interneurons (with permission from Sava et al., 2014).

Taurine activates cortical neurons via glycine receptors at a rather low affinity with an EC_50_ of about 1 mM (Flint et al., 1998; Okabe et al., 2004; **Figure 2B**). While no evidences for a synaptic activation of glycine receptor were found, inhibition of taurine transport with GES, hypoosmotic stimuli, and strong electrical stimulation evoke a putative nonsynaptic release of taurine, which activated glycine and probably also GABA_A_ receptors (Flint et al., 1998; Furukawa et al., 2014; Qian et al., 2014). Also in cortical neurons, the activation of glycine receptors mediates an excitatory effect, as concluded from taurine-induced Ca^2+^ transients and the fact that focal glycine application enhances the frequency of GABAergic PSCs (Flint et al., 1998). Sava et al. (2014) were able to replicate these findings and found that a prolonged application of 300 μM taurine induced a tonic inward current in putative projection neurons of the CP, which was in about 50% of the cells associated with a massive increase in GABAergic PSCs (**Figure 4A**). Pharmacological experiments revealed that about 80% of the tonic taurine-induced currents in these neurons are mediated via glycine, and about 20% via GABA_A_ receptors (Sava et al., 2014). The taurine-induced GABAergic responses are excitatory (**Figure 4B**) and are abolished in the presence of the glycinergic antagonist strychnine. As similar GABAergic PSCs could also be evoked by tonic application of glycine (Sava et al., 2014), it is concluded that glycine receptors on the presynaptic cells are essential to generate the GABAergic PSCs. Further analysis of the presynaptic GABAergic interneurons, which were visually identified in GAD67-GFP transgenic animals (Tamamaki et al., 2003), demonstrated that taurine induces a strychnine-sensitive inward current (**Figure 4C**), which was associated with a massive increase in action potential discharges in the majority of GABAergic interneurons (**Figure 4D**; Sava et al., 2014). This observation demonstrates that taurine indeed excites GABAergic interneurons. Further experiments revealed that these taurine-induced GABAergic PSCs increase the frequency of action potential discharges in putative pyramidal neurons (Sava et al., 2014). In summary, these studies demonstrated that taurine has an excitatory net effect in immature neuronal circuits *in situ* and thus most probably contribute network activity in the developing neocortex. However, it will be necessary to determine whether taurine contributes to excitation also *in vivo*, since recent experiments demonstrated depolarizing, but inhibitory effects of GABA_A_ receptors in the developing neocortex under *in vivo* conditions (Kirmse et al., 2015).

### Subplate Neurons

For SP neurons, it was also demonstrated that taurine activates glycine receptors with a low affinity (EC_50_ = 1.7 mM; Kilb et al., 2008). While higher taurine concentrations evoke desensitizing responses, 100 μM taurine induced a tonic inward current in this cell type (**Figure 2B**). Also in SP neurons, the activation of glycine receptors mediates a depolarizing action (Kilb et al., 2008). Further analyses revealed that tonic taurinergic currents, although evoking only small subthreshold depolarizations, substantially lower the action potential threshold, thus demonstrating an obvious excitatory effect on SP neurons. Inhibition of the taurine transport with GES as well as hypotonic stimulation induced inward currents, which suggests that also in the SP neurons taurine can be an endogenous agonist of glycine receptors (Kilb et al., 2008). In summary, these experiments indicate that taurine can act as neuromodulator in the SP. As a fraction of SP neurons are GABAergic, it was also investigated whether SP neurons contribute to the taurine-induced GABAergic PSCs in pyramidal neurons (see section “Neurons in the Cortical Plate or Developing Cortical Layers”). However, these experiments revealed that ablation of the SP does not significantly reduce the frequency of taurine-induced GABAergic PSCs, demonstrating that GABAergic projections from SP to pyramidal neurons do not considerably contribute to the taurine-induced activity in the CP (Sava et al., 2014). On the other hand, ambient taurine level could be highest in the embryonic SP (Furukawa et al., 2014). Since the SP is a crucial element for structural and functional development of the neocortex (for review Kanold and Luhmann, 2010) and is essentially involved in the regulation of excitability in the developing neocortex (Dupont et al., 2006; Hanganu et al., 2009), further experiments are needed to evaluate whether SP neurons are the particularly important target for the neurodevelopmental effects of taurine.

## Summary

**FIGURE 5:**
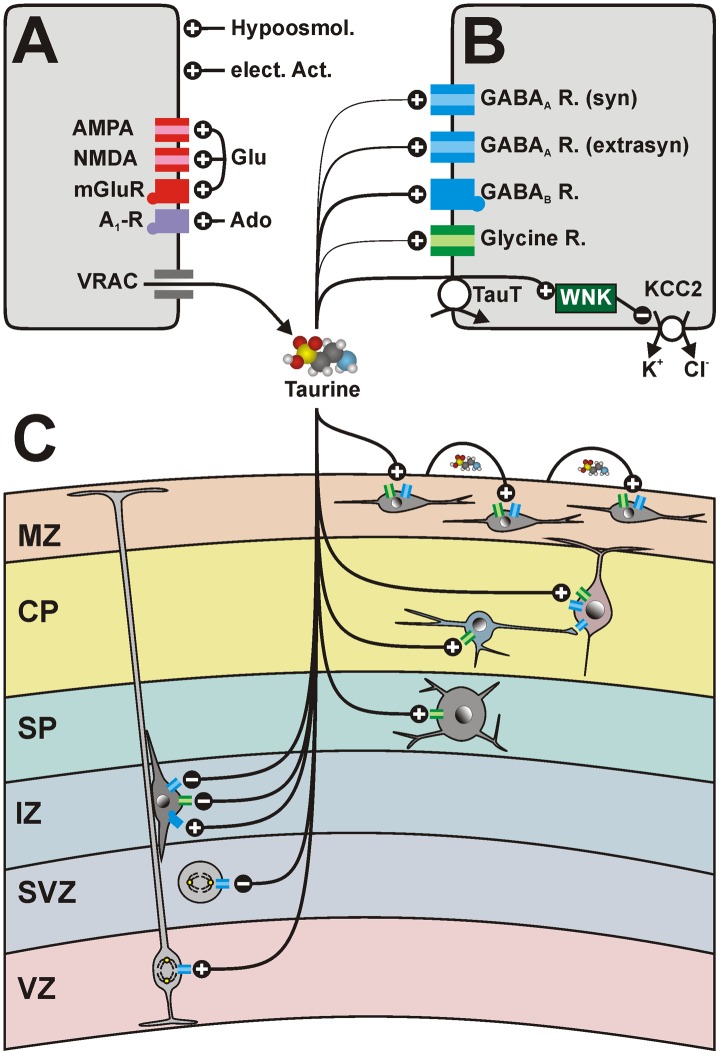
Schematic diagram summarizing the effects of taurine on the immature neocortex. **(A)** Taurine release is mediated mainly by volume-regulated anion channels (VRAC). The release of taurine is activated by hypoosmotic conditions, electrical activity and via glutamate (Glu), and adenosine (Ado) receptors. **(B)** Taurine mediates its effects via low-affinity binding to glycine receptors (green symbols) or GABA_A_ receptors (blue symbols) with subunit compositions typical for synaptic receptors. While the taurine affinity to putatively extrasynaptic GABA_A_ receptors is moderate, taurine is a high-affinity ligand for GABA_B_ receptors. In addition, the intracellular taurine concentration, regulated by the TauT, suppresses the function of the Cl^-^ extruder KCC2 via activation of the WNK pathway, thus maintaining depolarizing taurinergic membrane responses. **(C)** Putative effect of taurine on different cell populations in the developing neocortex. Taurine promotes proliferation in the VZ, but attenuates proliferation in the SVZ. It stimulates chemotaxis via GABA_B_ receptors and suppresses radial migration via GABA_A_ and glycine receptors. Taurine depolarizes SP neurons, pyramidal cell and GABAergic interneurons in the CP, as well as CRc in the MZ via activation of GABA_A_ and/or glycine receptors. The taurinergic depolarization of GABAergic interneurons is *in vitro* sufficient to generate GABAergic network activity transmitted to pyramidal cells. CRc participate to propagating activity in the MZ mediated by activity-dependent taurine release. See text for details.

Taurine can be considered as an important neurodevelopmental modulator (**Figure 5**). Taurine-mediated currents were identified on most major neuronal populations in the immature neocortex (Kilb, 2017). Due to its depolarizing effect on most investigated neuronal populations in the neocortex, taurine can directly induce intracellular Ca^2+^ transients, which are causal for many neurodevelopmental events like migration or differentiation (Spitzer et al., 2000; Komuro and Kumada, 2005). In addition, taurine increases neuronal excitability in most neocortical neuron types due to this depolarization. In accordance with this single-cell effects, it was shown that taurine increase the network activity in the immature CP *in vitro* (Sava et al., 2014). Taurine can therefore also indirectly impact neuronal development via these effects on activity patterns (for review Wang and Kriegstein, 2009; Luhmann et al., 2016). In the immature hippocampus, it has also be shown that nonsynaptic taurine can modulate neural excitability, dose dependently in both excitatory and inhibitory directions (Chen et al., 2014), due to a consistent depolarizing effect in combination with a dominant shunting effect at higher taurine concentrations. Selective inhibition of glycine receptors with strychnine induces in these immature hippocampal preparations epileptiform activity (Chen et al., 2014), indicating that taurine, as main endogenous agonist of glycine receptors in the immature CNS, most probably contribute to inhibitory net effects in the immature hippocampus. The next important step in the evaluation of taurinergic actions on the activity of immature networks would therefore be to unravel the direct effects of taurine under *in vivo* conditions, since in this situation activation of GABA_A_ receptors mediates depolarizing but inhibitory effects in the developing neocortex (Kirmse et al., 2015). Given that *in vitro* taurine mediates mainly excitatory network effects in the neocortex, but an inhibitory net action in the hippocampus, it would be interesting to uncover the effect of taurine on the excitability in other regions of the CNS, like thalamus, basal ganglia, hypothalamic nuclei, amygdala, and the spinal cord. The essential role of taurine for neuronal development entails that any interference with the taurinergic system during pregnancy or early childhood increases the risk for developmental disorders. Accordingly, the adverse effect of the antiepileptic drug vigabatrin, which severely impaired neuronal development (Manent et al., 2007), has already been correlated to a disturbed taurine homeostasis under these conditions (Jammoul et al., 2009).

## Author Contributions

WK and AF drafted, wrote, and revised the text. WK and AF approved the final version of the manuscript and agreed to be accountable for all aspects of the work in ensuring that questions related to the accuracy or integrity of any part of the work are appropriately investigated and resolved.

## Conflict of Interest Statement

The authors declare that the research was conducted in the absence of any commercial or financial relationships that could be construed as a potential conflict of interest.

## Abbreviations

BDNF: brain derived neurotrophic factor
bFGF: basic fibroblast growth factor
CNS: central nervous system
CP: cortical plate
CRc: Cajal–Retzius cells
GABA: gamma-aminobutyric acid
IZ: intermediate zone
MZ: marginal zone
PPL: primordial plexiform layer
PSCs: postsynaptic currents
SP: subplate
SVZ: subventricular zone
TauT: taurine transporter
VZ: ventricular zone
